# Soil centipedes (Chilopoda, Geophilomorpha) in the Val Camonica forests (Southern Alps): species composition and richness

**DOI:** 10.3897/BDJ.11.e103153

**Published:** 2023-05-29

**Authors:** Roberto Magnolini, Lucio Bonato

**Affiliations:** 1 Dipartimento di Biologia, Università di Padova, Padova, Italy Dipartimento di Biologia, Università di Padova Padova Italy; 2 National Biodiversity Future Centre, Palermo, Italy National Biodiversity Future Centre Palermo Italy

**Keywords:** Chilopoda, Southern Prealps, community ecology, species richness

## Abstract

Soil centipedes (Chilopoda, Geophilomorpha) are a widespread group of predators in the forest soils of the European Alps. While in the eastern and western parts of the Southern Prealps, larger efforts were devoted to sample and study the geophilomorph fauna, little is known about species richness and composition of geophilomorph communities in the central part of Southern Prealps. In this work, five sites located in the Val Camonica were surveyed by hand searching, between November 2021 and July 2022 and their species richness was estimated applying non-parametrical statistical methods (Chao-1 and Abundance-based Coverage Estimator) to account for incomplete detection. A total of 18 species were found amongst the five sites. A maximum of 12 species were recorded in each single site, while estimates suggest that another 1-3 species were likely undetected. Species composition were found highly variable also between sites with similar species richness.

## Introduction

Soil centipedes (Chilopoda Geophilomorpha) are a widespread component of the soil fauna (Jeffery et al. 2010, Orgiazzi et al. 2016). Geophilomorph communities of temperate forest soils are amongst the richest ones in the world (e.g. Petersen and Luxton (1982), Bonato et al. (2017)), even though tropical forests have been less studied. More than 10 species may co-exist in single sites of the European Alps and a total of 40 morphologically distinct species of have been recorded in this region (Bonato et al. 2014).

In comparison with other major groups of soil predators, many facets of the diversity of geophilomorph communities and their ecology are almost unknown (Bonato and Minelli 2009, Bortolin et al. 2018). Like many other soil invertebrates, geophilomorphs are strongly affected by local environmental factors. As a consequence, species composition and abundance of populations can change on a short spatial scale (e.g. Purchart et al. (2013)). In addition, most studies on selected communities reported only the number of species found, which is usually lower than the real number of species present in a given site, because of the well-known problem of incomplete species detection (Gotelli and Chao 2011). However, carrying out an exhaustive sampling is a hard task for geophilomorph centipedes: many species are almost completely endogeic and many are expected to perform seasonal migrations between soil strata to survive unfavourable environmental conditions (Voigtländer 2011). However, suitable methods of data analyses have been developed to overcome the problem of incomplete species detection (Gotelli and Chao 2011), but have been rarely applied to geophilomorph communities (e.g. Peretti and Bonato (2018)).

**Figure 1.**
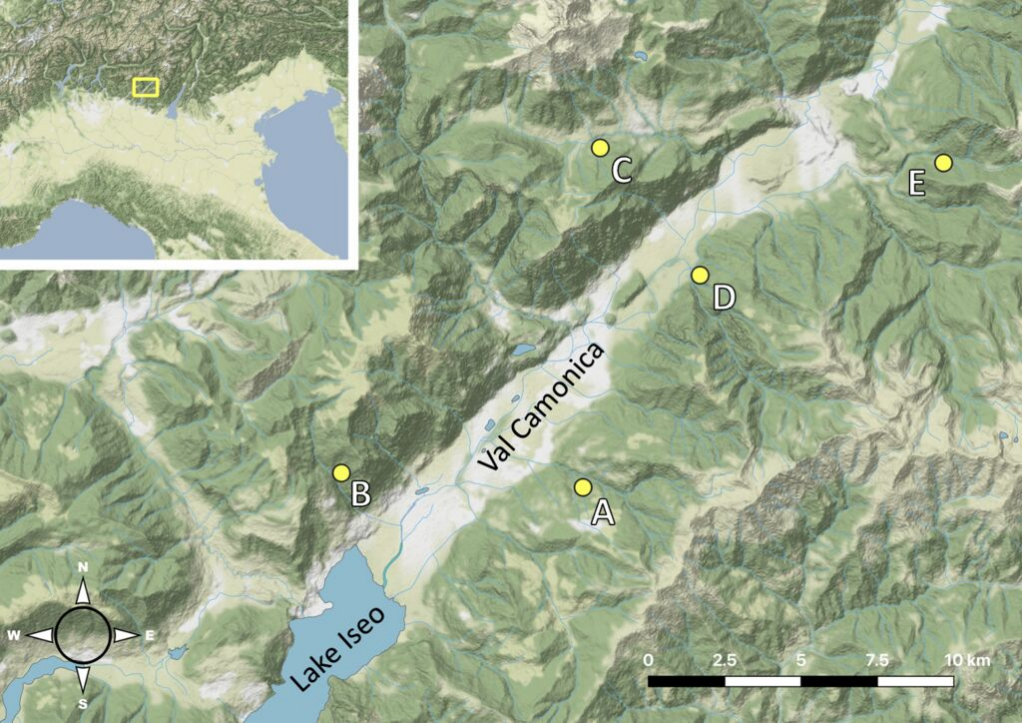
Sampling sites in the Val Camonica (yellow dots). Background from Stamen Design.

This paper presents the results of a survey of some geophilomorph communities in the forests of Val Camonica (Fig. 1). This area is located in the central part of the Southern Prealps, which is one of the least investigated areas within the European Alps. No targeted surveys have been carried out so far on geophilomorphs in the central part of the Southern Prealps, unlike the Western Prealps (Minelli and Zapparoli 1992) and the Eastern Prealps (Minelli 1987, Zapparoli 1989, Erhard 1996, Kos et al. 2015). Considering Val Camonica, only few records of geophilomorphs have been published so far: one record of *Eurygeophiluspinguis* (Brölemann, 1898) (Manfredi 1948), one of *Dicellophiluscarniolensis* (C.L. Koch 1847), two of *Geophilusimpressus* C.L. Koch, 1847 (recently adopted name for the species previously called *Geophilusalpinus* Meinert, 1870; see Popovici (2022)), one of *Himantariumgabrielis* (Linnaeus, 1767) (Zapparoli and Minelli 2005) and one of *Schendylacarniolensis* (Verhoeff, 1902) (Manfredi 1940).

The aims of the study were: (i) to contribute to filling the knowledge gap for the geophilomorph fauna of the Southern Prealps, by focusing on the Val Camonica forest soils and (ii) to estimate the species richness of selected communities with statistical models in order to adjust for incomplete detection.

## Material and Methods

### Study area

**Figure 2.**
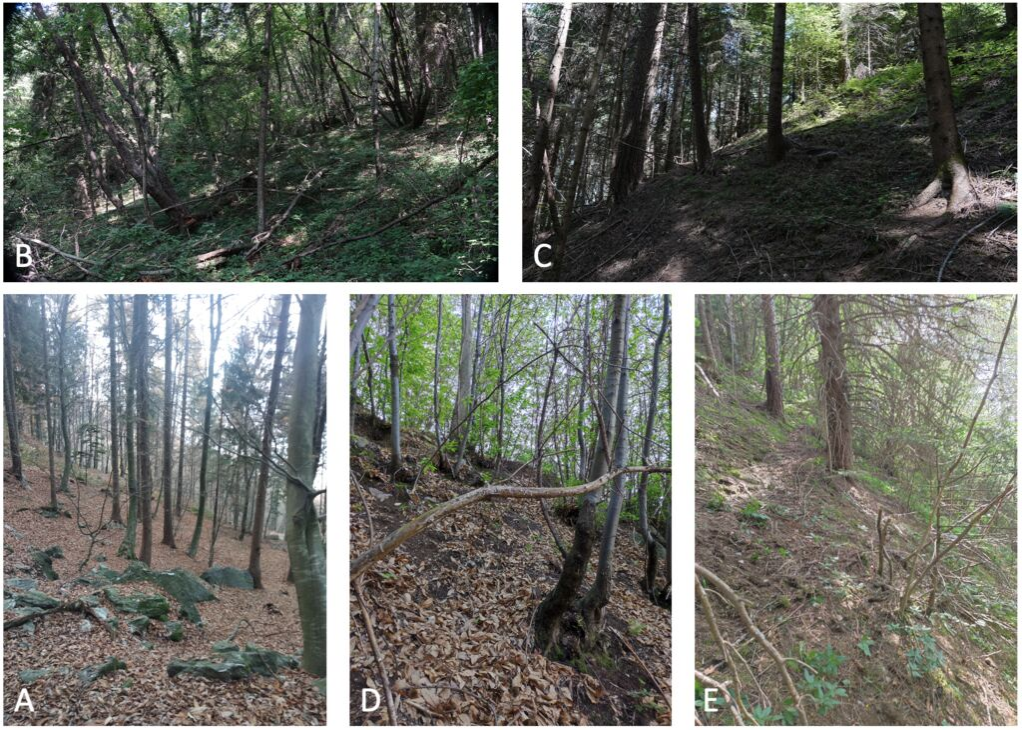
Sampling sites (see Table 1).

A total of five sites were studied in Val Camonica (Fig. 1, Table 1). The minimum distance between two sites was 5.3 km, while the maximum was 22.2 km. Sites were selected in forests, selecting those currently not affected by human usage other than wood harvesting (Fig. 2). Sites were chosen on both sides of the main valley.

Each site was defined as a circular area of radius 8 m, within a continuous forest patch of at least 0.25 ha, with uniform vegetation structure and at least 10 m away from forest edges, other ecotonal zones and roads.

### Sampling protocol

The five sites were visited between November 2021 and July 2022, for a total of 2-7 sampling sessions for each site (Suppl. material 1). Each sampling session was carried out for 1.0-1.5 hours by 1-4 researchers, who searched in parallel by hand on the ground, digging with a small shovel in the leaf litter and soil, digging deep to about 15 cm (when possible) and turning stones and rotten wood on the surface. This method was chosen because, in our experience, it is one of the most effective for both epigeic and endogeic centipedes, including geophilomorphs.

All specimens of geophilomorphs were collected in test tubes and fixed with 70% ethanol.

### Species identification

Specimens were identified to species level using a Leica DMLB microscope with magnification up to 400×, after mounting the specimens on temporary microscopic slides (Pereira 2000). When none of the two pretarsi of the second maxillae was visible, the head of the specimen was detached from the trunk (see Bonato et al. (2010), for anatomical terminology).

Species identification was conducted by means of Chilokey (Bonato et al. 2014) and, when necessary, considering the original descriptions or subsequent re-descriptions of the species. For taxonomy and nomenclature, the Checklist of the Italian Fauna was followed (Bonato and Minelli 2021).

### Species composition

Differences in species composition between sites were evaluated with the Jaccard similarity index, which is based on presence-absence data. A Correspondence Analysis was also performed in order to assess the pattern of diversity between sites. Since sites received different sampling efforts, the analysis was performed on presence-absence data, not on abundance data. The analysis was performed with the FactoMineR package in R (Husson et al. 2007, Lê et al. 2008) and biplots were generated with the Factoextra package in R (Greenacre 2010, Kassambara and Mundt 2017).

### Species richness estimation

The number of species in each site was estimated using two non-parametric estimators: the Chao-1 estimator, which is based on the proportion between the number of species collected once and the number of those collected twice (Chao 1984) and the Abundance-based Coverage Estimator (ACE), which is based on the frequency of “rare” species (Chao and Lee 1992). These estimators allow one to overcome the limitations of parametric estimators, which do not cope with the undersampling bias (Magurran 2004).

Chao-1 and ACE were calculated using PAST 4.08 (Hammer et al. 2001) and the vegan package in R (Oksanen et al. 2017) using all parameters as default; 95% confidence intervals were computed by the bootstrap method in PAST.

In order to compare species richness amongst sites, rarefaction and extrapolation were integrated from the numbers of detected species, with 95% confidence intervals based on “unconditional” variance, as proposed by Colwell et al. (2012). The analysis was performed with the iNEXT package in R (Hsieh et al. 2020), which uses the bootstrap method proposed by Chao et al. (2014). The parameters were set as default, except for the number of permutations, which was set to 150. A rarefaction analysis with 95% confidence intervals, based on “conditional” variance (Magurran 2004), was also performed with PAST.

## Results

A total of 38 hours of sampling sessions allowed us to collect 242 specimens. Between 31 and 85 specimens were collected per site. All specimens were identified to species level, for a total of 18 species detected (Table 2).

### Species composition

Considering the species detected in the five communities, the pairwise values of the Jaccard similarity index were between 0.11 (between sites D and E) and 0.38 (between sites B and C), with a mean value of 0.26 (Table 3).

**Figure 3.**
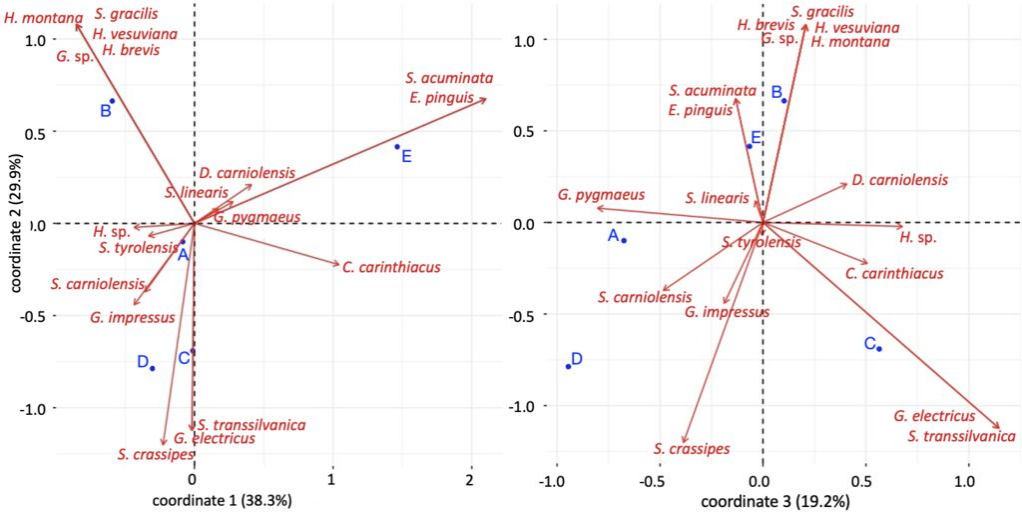
Contribution biplots of the Correspondence Analysis performed on the presence-absence of species in five sites in Val Camonica. Red arrows correspond to species, blue dots correspond to sites.

The Correspondence Analysis performed on presence-absence data produced three main coordinates, accounting for 38%, 30% and 19% of the total variance, respectively (Fig. 3). Taking into account the first two coordinates, community E was different from all other sites because of the presence of *Strigamiaacuminata* and *Eurygeophiluspinguis*, while community B separated from all the others because of the presence of a probably undescribed species of *Geophilus*, *Heniavesuviana*, *H.montana*, *H.brevis* and *Stigmatogastergracilis*. The communities C and D differed from the others and shared the presence of *S.crassipes* and *G.impressus*. The third coordinate also allowed us to distinguish community A from most of the others.

### Species richness

**Figure 4.**
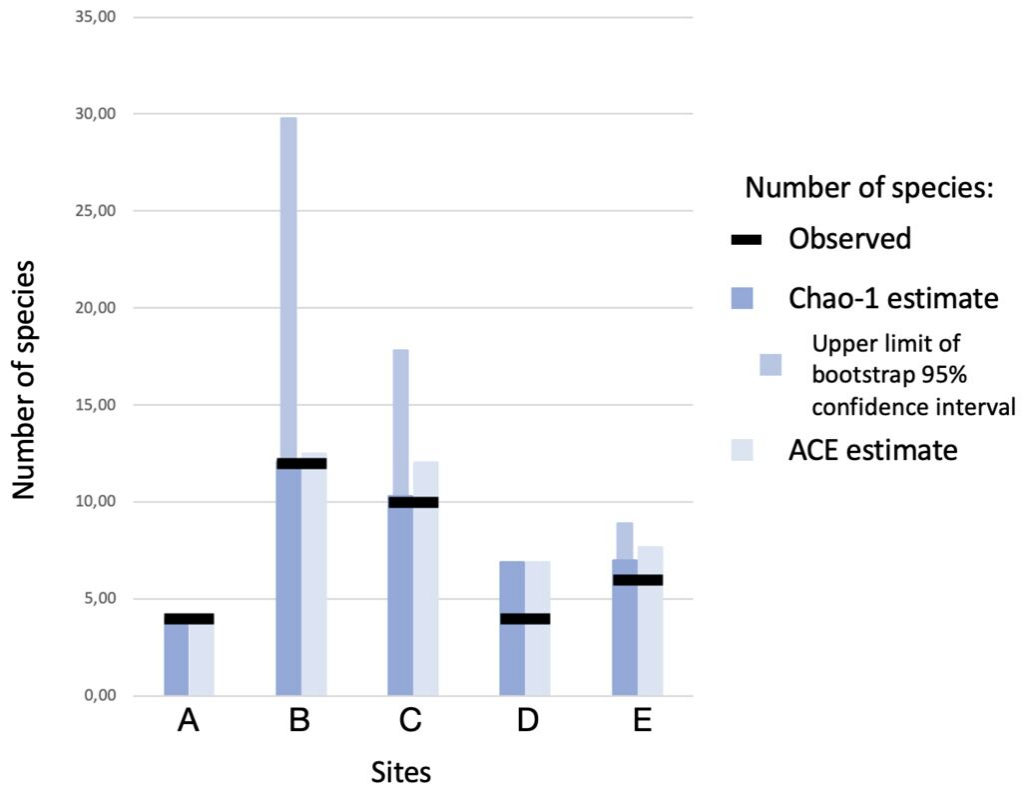
Observed and estimated species richness of Geophilomorpha in five sites in Val Camonica.

Between 4 and 12 species were detected in each of the five sites (Fig. 4, Table 4): 4-6 in three sites (A, D, E) and 10-12 in the other two (B, C). In most of the sites, estimates of species richness (Chao-1 and ACE) exceeded the observed number of species, with 1-3 species likely undetected (Fig. 4, Table 4). In the sites with the highest number of observed species (B and C), the estimators suggested that the sampling was pretty exhaustive, but the 95% confidence intervals of Chao-1 indicated the possibility of many other undetected species (Fig. 4, Table 4). PAST and vegan gave very similar results. The two sites with the highest species richness (B and C) were also the two most similar to each other (see Table 3).

**Figure 5.**
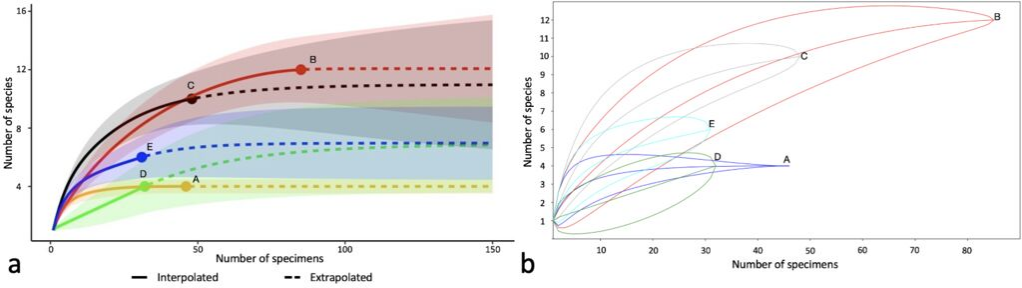
Comparison of the estimated species richness of Geophilomorpha amongst five sites in Val Camonica. **a** Rarefaction curves (solid lines) and extrapolated curves (dashed lines), with 95% confidence intervals based on unconditional variance (coloured areas). **b** Rarefaction analysis, with 95% confidence intervals based on conditional variance.

The rarefaction analysis with 95% confidence intervals, based on “unconditional” variance (Fig. 5a), indicated a statistically significant difference in species richness between the poorest site (A, with four detected species and no estimated undetected species) and the sites B, C and E. Moreover, the rarefaction analysis with 95% confidence intervals, based on “conditional” variance (Fig. 5b), suggested that sites B and C are significantly richer than site D.

## Discussion

This study provides the first insights on species richness and composition variation of the geophilomorph communities living in the forests of Val Camonica. Therefore, it contributes to fill a gap in the knowledge of the geophilomorph fauna of the central sector of the Southern Prealps, which has been poorly investigated up to date (see above in Introduction). Only two species had been already previously recorded in Val Camonica, namely *Dicellophiluscarniolensis* and *Eurygeophiluspinguis*, while another 16 species were found anew in the area (Table 2). Amongst these species, two are most probably still undescribed and belong to the genera *Henia* C.L. Koch, 1847 and *Geophilus* Leach, 1814. All the other species found were expected, because they had been already reported from the montane areas both west and east of Val Camonica, i.e. from the Bergamasque Alps and Prealps in the west and from the Brescia the Garda Prealps in the east (Zapparoli 1989, Minelli and Zapparoli 1992; Table 5).

On the other hand, three species, previously recorded in Val Camonica, were not found in the five studied sites, namely *Geophilusflavus, Pachymeriumferrugineum* and *Himantariumgabrielis*. Especially the latter is expected to be strictly limited to xerothermic sites along the Southern Prealps (Zapparoli and Minelli 2005).

Other species reported from contiguous areas were not found in Val Camonica (Table 5) Regarding *Strigamiaengadina*, it should be noted that the taxonomic value of this species is still uncertain because the morphology is inadequately known (Bonato and Minelli 2014). Moreover, records of *S.engadina* from the Brescia Prealps are probably due to misidentification, according to Bonato and Minelli (2021). *Geophiluscarpophagus* has been frequently recorded in the past along the Sourthern Prealps (Zapparoli and Minelli 2005). However, its actual occurrence as an indigenous species needs confirmation. A similar explanation may be provided for the single old record of *G.osquidatum* from the Southern Prealps (Bonato and Minelli 2021). On the other hand, the apparent absence of *Clinopodesflavidus* and *Pleurogeophilusmediterraneus* from Val Camonica is notable because especially the former species is known to be spread both on the Bergamasque Prealps (Zapparoli and Minelli 2005) and on the Garda Prealps (Minelli 1992).

### Species composition and relationship with species richness

There are few studies that compare local communities of geophilomorphs in terms of species richness (e.g. Grgič and Kos 2005, Leśniewska et al. 2005, Leśniewska and Leśniewski 2015, Peretti and Bonato 2018). In Val Camonica, geophilomorph communities with similar estimates of number of species (4-7) have actually very different composition (as shown in Table 3). Additionally, the other two communities with more numerous species estimates (10-13) have different composition (only five species in common). These differences could be explained by habitat differences (Table 1), but more studies are needed to understand which ecological parameters have the greatest influence on the composition of geophilomorph communities.

Results of this work could be affected by some methodological limits. The estimates of species richness and their comparisons between sites could be biased by different probability of detection between species and between different sites for the same species. Despite this, the hand-searching method adopted by us permitted us to maximise the sampling rate of geophilomorphs and to also capture strictly endogeic species, unlike other commonly employed methods (e.g. pitfall traps), as also shown by Tuf (2015).

### Richness estimates

Real data as well as non-parametric estimators indicate that more than 12 species of geophilomorphs – not considering high level of uncertainty because of large confidence intervals – can regularly live in syntopy in the study area (Fig. 4).

Considering the Southern Prealps and Dinarides, a few other studies estimated centipede species richness using statistical tools to account for incomplete detection (Grgič and Kos 2003, Grgič and Kos 2005, Peretti and Bonato 2018; Table 6). However, these studies did not provide separate estimates for the geophilomorphs alone. Taking into account the absolute number of detected species, between 4 and 16 species of geophilomorphs were found co-existing, amongst all the studied sites, with a mean of 9-10 species. On the other hand, the five sites sampled in Val Camonica have a lower mean of detected species (7).

## Supplementary Material

9B9A839A-DD89-5D6F-B4DE-F15EF1AD44E3 10.3897/BDJ.11.e103153.suppl1 7989363 Supplementary material 1 Sampling sessions for Geophilomorpha in five sites of Val Camonica Data type dates and time spent in each sampling session. File: oo_841004.xlsx https://binary.pensoft.net/file/841004 Magnolini Roberto, Bonato Lucio

## Figures and Tables

**Table 1.** Geographic features of the sampling sites in Val Camonica. Lithological data are from Bucci et al. (2022). Climatic data are from Wessely et al. (2022) and refer to the period 1970-2005 for precipitations and 1950-2000 for temperatures. All other data have have been take directly in the field.

**Site**	**Latitude** **(°N)**	**Longitude** **(°E)**	**Altitude** **(m)**	**Aspect**	**Lithological** **substrate**	**Dominant tree species**	**Mean annual** **precipitation** **(mm/year)**	**Mean annual** **temperature** **(°C)**
A	45.8368	10.1812	760	N	Schistose metamorphic	*Castaneasativa*, *Larixdecidua*, *Piceaabies*	1162	9.9
B	45.8420	10.0795	695	SW	Carbonate	*Castaneasativa*, *Ostryacarpinifolia*, *Piceaabies*, *Quercuspetraea*	1178	10.3
C	45.9366	10.1906	1070	WNW	Carbonate and mixed sedimentary	*Abiesalba*, *Piceaabies*	1346	7.8
D	45.8987	10.2319	525	WNW	Siliciclastic sedimentary	*Castaneasativa*, *Fagussylvatica*	964	11.0
E	45.9306	10.3354	1190	NNW	Glacial drift	*Corylusavellana*, *Fagussylvatica*, *Larixdecidua*, *Piceaabies*	1343	7.5

**Table 2.** Species of Geophilomorpha and number of specimens found in five sites in Val Camonica. Families after Bonato et al. (2013). * Putative undescribed species.

	**Sites**					**All sites**
	**A**	**B**	**C**	**D**	**E**	
**Geophilidae**						
*Clinopodescarinthiacus* (Latzel, 1880)	-	-	3	-	4	7
*Eurygeophiluspinguis* (Brölemann, 1898)	-	-	-	-	1	1
*Geophiluselectricus* (Linnaeus, 1758)	-	-	1	-	-	1
*Geophilusimpressus* C.L. Koch, 1847	-	2	3	1	-	6
*Geophiluspygmaeus* Latzel, 1880	23	54	-	29	4	110
*Geophilus* sp.*	-	2	-	-	-	2
*Heniabrevis* (Silvestri, 1896)	-	6	-	-	-	6
*Heniamontana* (Meinert, 1870)	-	1	-	-	-	1
*Heniavesuviana* (Newport, 1845)	-	2	-	-	-	2
*Henia* sp.*	-	3	2	-	-	5
*Stenotaenialinearis* (C.L. Koch, 1835)	3	7	12	-	13	35
*Strigamiaacuminata* (Leach, 1815)	-	-	-	-	1	1
*Strigamiacrassipes* (C.L. Koch, 1835)	-	-	1	1	-	2
*Strigamiatranssilvanica* (Verhoeff, 1928)	-	-	3	-	-	3
**Himantariidae**						
*Stigmatogastergracilis* (Meinert, 1870)	-	2	-	-	-	2
**Mecistocephalidae**						
*Dicellophiluscarniolensis* (C.L. Koch, 1847)	-	2	10	-	8	20
**Schendylidae**						
*Schendylacarniolensis* Verhoeff, 1902	13	2	11	1	-	27
*Schendylatyrolensis* (Meinert, 1870)	7	2	2	-	-	11
**Total specimens**	46	85	48	32	31	242

**Table 3.** Jaccard similarity index amongst five sites in Val Camonica.

**Sites**	**A**	**B**	**C**	**D**	**E**
**A**	-	0.33	0.27	0.33	0.25
**B**	0.33	-	0.38	0.23	0.20
**C**	0.27	0.38	-	0.27	0.23
**D**	0.33	0.23	0.27	-	0.11
**E**	0.25	0.20	0.23	0.11	-

**Table 4.** Observed and estimated values of species richness of Geophilomorpha in five sites in Val Camonica.

	**Sites**				
	**A**	**B**	**C**	**D**	**E**
**Observed species**	4	12	10	4	6
**Estimated richness by Chao-1**	4.00	12.07	10.33	6.91	6.97
**Upper limit of the 95% confidence interval of Chao-1 index (9999 bootstrap replicates)**	4.00	29.79	17.83	6.91	8.90
**Estimated richness by ACE**	4.00	12.50	12.05	6.91	7.68

**Table 5.** Species of Geophilomorpha recorded in Val Camonica and neighbouring sections of the Alps (boundaries according to Marazzi (2005)). Data from the Zapparoli and Minelli (2005) and subsequent publications.

	**Bergamasque Alps** **and Prealps**	**Val Camonica**	**Southern** **Rhaetian Alps**	**Brescia and** **Garda Prealps**
*Clinopodescarinthiacus* (Latzel, 1880)	X	X	-	X
*Clinopodesflavidus* C.L. Koch, 1847	X	-	?	X
*Dicellophiluscarniolensis* (C.L. Koch, 1847)	X	X	X	X
*Dignathodonmicrocephalus* (Lucas, 1846)	-	-	-	X
*Eurygeophiluspinguis* (Brölemann 1898)	X	X	-	X
*Geophiluscarpophagus* Leach, 1815	X	-	-	X
*Geophiluselectricus* (Linnaeus, 1758)	-	X	-	X
*Geophilusflavus* (De Geer, 1778)	X	X	-	X
*Geophilusimpressus* C.L. Koch, 1847	X	X	X	X
*Geophilusosquidatum* (Brölemann 1909)	?	-	-	-
*Geophiluspygmaeus* Latzel, 1880	X	X	X	X
*Geophilus* sp.	-	X	-	-
*Heniabrevis* (Silvestri, 1896)	X	X	-	X
*Heniamontana* (Meinert, 1870)	X	X	X	X
*Heniavesuviana* (Newport, 1845)	X	X	X	X
*Henia* sp.	?	X	-	-
*Himantariumgabrielis* (Linnaeus, 1767)	X	X	-	X
*Pachymeriumferrugineum* (C.L. Koch, 1835)	X	X	?	X
*Pleurogeophilusmediterraneus* (Meinert, 1870)	X	-	-	X
*Schendylacarniolensis* Verhoeff, 1902	X	X	X	X
*Schendylanemorensis* (C.L. Koch, 1837)	X	-	?	-
*Schendylatyrolensis* (Meinert, 1870)	X	X	X	X
*Stenotaenialinearis* (C.L. Koch, 1835)	X	X	-	X
*Stigmatogastergracilis* (Meinert, 1870)	X	X	X	X
*Strigamiaacuminata* (Leach, 1815)	X	X	X	X
*Strigamiacrassipes* (C.L. Koch, 1835)	X	X	X	X
*Strigamiaengadina* (Verhoeff, 1935)	X	-	-	?
*Strigamiatranssilvanica* (Verhoeff, 1928)	X	X	X	X

**Table 6.** Number of species present in geophilomorph communities of the Southern Prealps, from studies based on high sampling efforts and statistical analyses accounting for incomplete detection.

**Source**	**Sector**	**Site**	**Number of species detected**	**Number of specimens detected**
Current paper	Brescia and Garda Prealps	A = Acquebone: near Ca' de Gos	4	46
	Bergamasque Alps and Prealps	B = Stramazzano: Torrente Supine	12	85
	Bergamasque Alps and Prealps	C = Borno: under Fienili Mensi	10	48
	Brescia and Garda Prealps	D = Sacca: Valle del Resio	4	32
	Southern Rhaetian Alps	E = Passo Crocedomini: over Degna	6	31
Grgič and Kos (2003)	Dinaric Alps	Near Iska, south of Ljubljana	16	-
Grgič and Kos (2005)	Dinaric Alps	Kumrova Vas	4	-
	Dinaric Alps	Mala Gora	10	-
	Dinaric Alps	Zeljne	8	-
	Dinaric Alps	Somova Gora	12	-
Peretti and Bonato (2018)	Dolomites	A = Costagranda: Ponte dei Ross	11	126
	Dolomites	B = Val del Mis: California	12	75
	Dolomites	C = Maragno	10	50
	Dolomites	D = Monte Tamberella	10	37
	Dolomites	E = Pian d'Avena	9	63
	Dolomites	F = Lago della Stua	11	58
	Dolomites	G = Val Pegolera	9	66
	Dolomites	H = Caiada: Casera d'Igoli	7	26
	Dolomites	I = Maragno	8	25
	Dolomites	J = Le Boscaie	6	26
